# Methylglyoxal-Stimulated Mesothelial Cells Prompted Fibroblast-to-Proto-Myofibroblast Transition

**DOI:** 10.3390/ijms26020813

**Published:** 2025-01-19

**Authors:** Yu-Syuan Wei, Su-Yi Tsai, Shuei-Liong Lin, Yi-Ting Chen, Pei-Shiue Tsai

**Affiliations:** 1 Graduate Institute of Veterinary Medicine, School of Veterinary Medicine, National Taiwan University, Taipei 10617, Taiwan; f08629016@ntu.edu.tw; 2 Department of Veterinary Medicine, School of Veterinary Medicine, National Taiwan University, Taipei 10617, Taiwan; 3 Department of Life Science, College of Life Science, National Taiwan University, Taipei 10617, Taiwan; suyitsai@ntu.edu.tw; 4 Research Center for Developmental Biology and Regenerative Medicine, National Taiwan University, Taipei 10617, Taiwan; linsl@ntu.edu.tw; 5 Graduate Institute of Physiology, College of Medicine, National Taiwan University, Taipei 10051, Taiwan; 6 Department of Internal Medicine, National Taiwan University Hospital, Taipei 10002, Taiwan; chenyiting@ntuh.gov.tw; 7 Department of Integrated Diagnostics & Therapeutics, National Taiwan University Hospital, Taipei 10002, Taiwan

**Keywords:** proto-myofibroblast, peritoneal fibrosis, methylglyoxal, fibroblast-to-myofibroblast, mesothelium

## Abstract

During long-term peritoneal dialysis, peritoneal fibrosis (PF) often happens and results in ultrafiltration failure, which directly leads to the termination of dialysis. The accumulation of extracellular matrix produced from an increasing number of myofibroblasts was a hallmark characteristic of PF. To date, glucose degradation products (GDPs, i.e., methylglyoxal (MGO)) that appeared during the heating and storage of the dialysate are considered to be key components to initiating PF, but how GDPs lead to the activation of myofibroblast in fibrotic peritoneum has not yet been fully elucidated. In this study, mesothelial cell line (MeT-5A) and fibroblast cell line (MRC-5) were used to investigate the transcriptomic and proteomic changes to unveil the underlying mechanism of MGO-induced PF. Our transcriptomic data from the MGO-stimulated mesothelial cells showed upregulation of genes involved in pro-inflammatory, apoptotic, and fibrotic pathways. While no phenotypic changes were noted on fibroblasts after direct MGO, supernatant from MGO-stimulated mesothelial cells promoted fibroblasts to change into proto-myofibroblasts, activated fibroblasts in the first stage toward myofibroblasts. In conclusion, this study showed that MGO-stimulated mesothelial cells promoted fibroblast-to-proto-myofibroblast transition; however, additional involvement of other factors or cells (e.g., macrophages) may be needed to complete the transformation into myofibroblasts.

## 1. Introduction

Fibrosis, which is characterized by excessive accumulation of extracellular matrix (ECM) in injured tissues, has drawn great attention because of its detrimental impacts on both the architecture and function of a variety of organs. Chronic inflammation induced by various stimulants is the most dominant cause of developing tissue fibrosis [1,2]. While the appearance of myofibroblasts was considered the hallmark circumstance during the fibrotic process, the involvement of different types of cells and complex cell–cell interactions should also be considered [3,4]. Myofibroblasts, a type of mesenchymal cell, appear upon the tissue remodeling process and exhibit prominent ECM secretion ability [5,6], and it is known that fibrosis of different organs may have distinct origins of myofibroblasts. To date, several cell types, such as epithelial cells, macrophages, or fibroblasts, had all been postulated as the potential progenitor cells of myofibroblasts; however, the most accepted consensus on myofibroblast precursors was still the resident fibroblasts [7,8,9,10,11]. During the transition of fibroblasts to myofibroblasts, the fibroblasts undergo a series of changes, such as forming stress fibers and adhesion complexes, increasing ECM protein production, and obtaining strong contraction ability [11,12]. Interestingly, during the process of fibroblast to myofibroblast transition, an intermediate phenotype of activated fibroblasts that showed stress fibers and active ECM synthesis ability without the presence of alpha-smooth muscle actin (αSMA), a typical hallmark of myofibroblasts, had also been described and named proto-myofibroblasts [6,12]. By detecting focal adhesion complex, actin filament bundles, and extra domain A containing fibronectin (EDA-FN), proto-myofibroblasts could be distinguished from quiescent fibroblasts [13,14].

Peritoneal fibrosis is a common complication occurring in long-term peritoneal dialysis (PD) patients and can ultimately lead to the termination of the dialysis. A healthy peritoneum is composed of a single layer of mesothelial cells and a sub-mesothelial zone having a gel-like matrix with fibroblasts and a network of capillaries. During long-term PD, under repetitive stimulations of glucose in high concentration and glucose-degradation products (GDPs) from the dialysate, the peritoneum undergoes pathological changes such as abnormal ECM accumulation, loss of mesothelial cells, increased number of myofibroblasts, and angiogenesis [15,16,17]. Although various mediators that participate in the occurrence of peritoneal fibrosis have been described, studies that focus on investigating the relationships and communications between peritoneal cell populations are limited [18,19].

In this study, we utilized methylglyoxal (MGO), a major component of GDPs and a powerful inducer of peritoneal fibrosis in various animal models [20,21,22,23], as the stimulant to mimic the peritoneal injury resulting from dialysate and interrogate the role of mesothelial cells upon the transition of fibroblasts to myofibroblasts under an in vitro system.

## 2. Results

### 2.1. Direct MGO Stimulation Did Not Induce Fibroblast-to-Myofibroblast Transition

**Figure 1:**
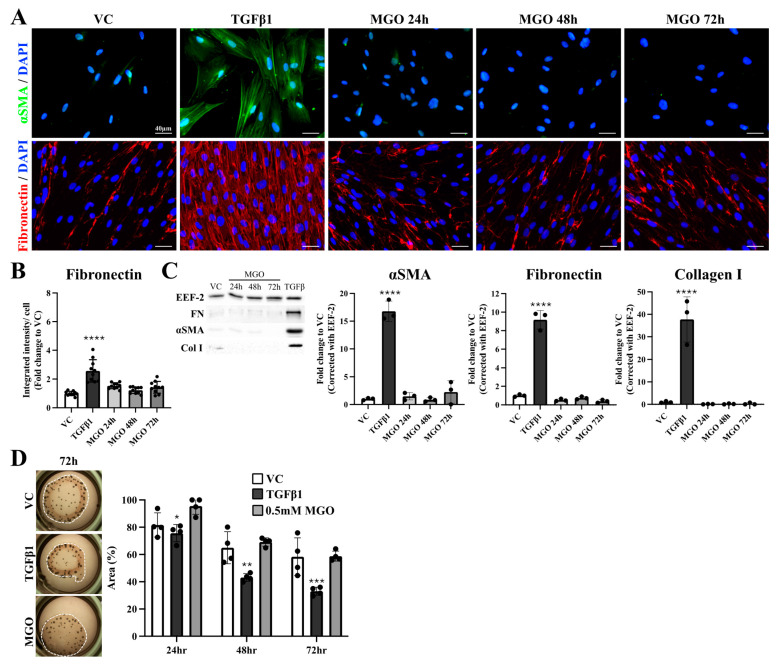
Examination of myofibroblast activation on MGO-treated fibroblasts. (**A**) Immunofluorescence results showed a significantly increased expression of αSMA (labeled in green) as well as fibronectin (labeled in red) in cells from the TGFβ1 group but not in that from the MGO groups. (**B**) Quantification analyses of immunofluorescence staining showed a 2-more fold increase in fibronectin expression in cells from the TGFβ1 group compared with the VC group. No differences were observed between the MGO-treated groups and the VC group. (**C**) Results of the Western blot showed that only cells treated with TGFβ1 induced a significant increase in protein expression of αSMA, fibronectin, and collagen I. (**D**) Collagen assay showed a significant shrinkage of fibroblast-laden collagen gels in the TGFβ1 group when compared with the VC group at all time points. However, treatment of 0.5 mM MGO causes no differences regarding the area of collagen gels, indicating that MGO-treated fibroblasts shared similar contraction ability with normal fibroblasts. Asterisks indicated significant differences between groups (**** *p* < 0.0001, *** *p* < 0.001, ** *p* < 0.01, * *p* < 0.05). Images from 10 different fields in each group were evaluated, and representative images were presented.

To investigate whether MGO directly arouses the activation of fibroblasts, multiple concentrations of MGO were co-incubated with MRC-5 for various time periods, and an MTT assay was performed to evaluate the cytotoxicity of MGO on fibroblasts. As shown in Supplementary Figure S1A, 0.5 mM MGO was selected for the following experiments as it is the highest concentration that did not cause significant cytotoxicity within 72 h (Supplementary Figure S1A). Since TGFβ1 had been shown to activate the conversion from fibroblasts into myofibroblasts [24,25], TGFβ1 was used as the positive control group in this study. Protein expression of αSMA, the hallmark protein of myofibroblasts, and fibronectin, an important member of ECM, were investigated through immunofluorescence staining first. As shown in Figure 1A, increased αSMA signals with rigid and straight lines in the cytosolic pattern from the TGFβ1-treated group were observed, while no apparent signals were from cells of the VC group and MGO-treated groups (Figure 1A, upper panel). As for fibronectin, sparse signals were detected in the VC group and MGO groups, while intense signals were observed in the TGFβ1-treated group (Figure 1A, lower panel; Supplementary Figure S3). The quantification results showed significantly increased signals of fibronectin in the TGFβ1-treated group when compared to that in the VC group, while no differences were noted between cells from the VC group and MGO groups (Figure 1B). To confirm the above-mentioned observation, myofibroblast marker proteins such as αSMA, fibronectin, and collagen I were also evaluated using Western blot. Similar to the immunofluorescent staining results, a significant increase in protein expressions for αSMA, fibronectin, and collagen I can be detected in TGFβ1-treated fibroblasts, but no alterations were noted in cells from MGO-treated groups when compared with the VC group (Figure 1C). Since myofibroblasts usually exhibit stronger contraction ability than regular fibroblasts, the examination of contraction ability was further conducted. A significantly decreased area of MRC-5-laden collagen gel was observed after 24-72 h of TGFβ1 stimulations when compared with the VC group, which indicated that TGFβ1-treated fibroblasts have increased contraction ability (Figure 1D). However, the area of gels showed no differences between the VC group and MGO-treated groups. These results suggested that direct MGO stimulation on fibroblasts did not activate fibroblast-to-myofibroblast transition on the basis of marker protein expression, ECM protein expression, and contraction ability.

### 2.2. Transcriptomic Analysis Revealed a Time-Dependent Activation of Inflammatory Pathways in MGO-Treated Mesothelial Cells

**Figure 2:**
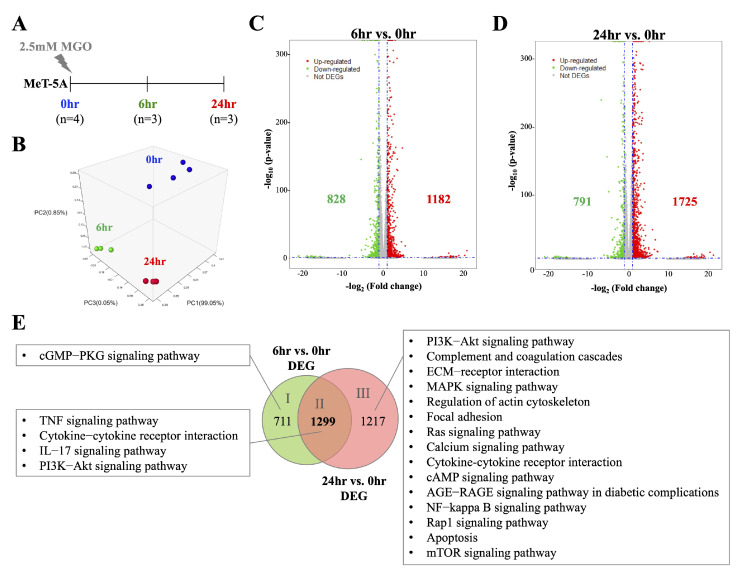
NGS results of MGO-stimulated mesothelial cells revealed distinct transcriptomic signatures between different treating times. (**A**) Experimental design of the samples that were sent to perform NGS analyses. (**B**) Distinct characteristics of transcriptome can be seen in cells among different MGO-treated times by PCA. Samples collected at time points of 0 h, 6 h, and 24 h were labeled in blue, green, and red colors, respectively. (**C**) 1182 upregulated DEGs and 828 downregulated DEGs were detected when comparing transcriptomic profiles at the time point of 6 h with 0 h. (**D**) 1725 upregulated DEGs and 791 downregulated DEGs were detected when comparing transcriptomic profiles at the time point of 24 h with 0 h. (**E**) Venn diagram showed that more than half of the DEGs from “6 h vs. 0 h” and “24 h vs. 0 h” were overlapped. DEGs from areas I, II, and III were sent to analyze the corresponding pathways, and the significant pathways (*p* < 0.05) were listed respectively.

As mesothelium is the first layer of the peritoneum that is exposed to the dialysate, the response and secretions of mesothelial cells under the stimulation of GDPs were considered critical to initiate peritoneal fibrosis. To obtain a comprehensive profile of how mesothelial cells reacted and changed under the stimulation of MGO, MeT-5A treated with 2.5 mM MGO for 0, 6, and 24 h were collected, and their transcriptomic changes were analyzed through NGS (Figure 2A). The concentration of MGO applied on mesothelial cells (MeT-5A) was selected based on MTT and DCFH-DA assays (Supplementary Figure S1B,C). Under the treatment of 2.5 mM MGO, the mesothelial cells showed no significant changes in cell viability but demonstrated a significant appearance of oxidative stress, indicating the effects of MGO. Sequencing data were analyzed using PCA to assess the overall discrepancy in transcriptome between individual samples, and distinct clusters between different time points were observed (Figure 2B). As illustrated in volcano plots, more DEGs were noted when compared between 24 h vs. 0 h and 6 h vs. 0 h, indicating a time-dependent increase in transcriptomic difference was detected after MGO stimulation (Figure 2C,D). Approximately 1299 DEGs constantly appeared throughout the treatment and were further analyzed, and relevant biological pathways were also indicated. As shown in Figure 2E, pro-inflammatory pathways such as the tumor necrosis factor (TNF) signaling pathway, cytokine–cytokine receptor interaction, interleukin-17 (IL-17) signaling pathway, and PI3K-Akt signaling pathway were the main activated signaling pathways in MeT-5A throughout the MGO stimulation (Figure 2E). It is worth noting that more pro-inflammatory and fibrosis-related pathways and their corresponding DEGs were observed in the 24 h (area III) group, suggesting the tendency toward fibrosis.

### 2.3. Genes Involved in Pro-Inflammatory and Fibrosis-Related Pathways Were Significantly Upregulated upon MGO Stimulation on Mesothelial Cells

**Figure 3:**
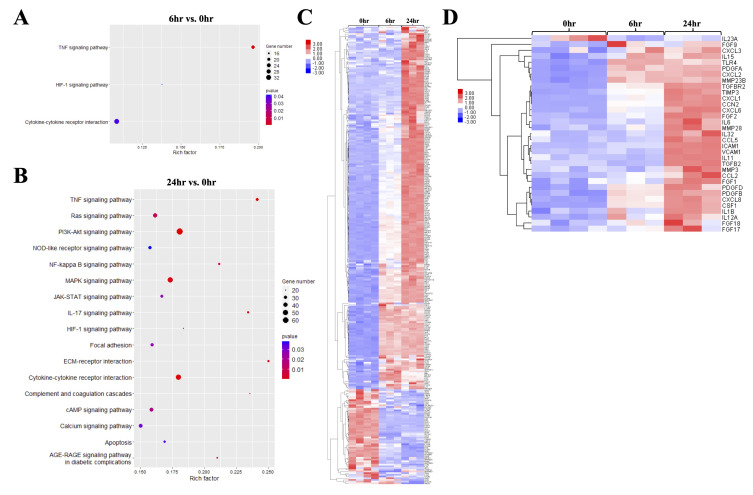
Pathways associated with inflammation and fibrosis were significantly altered upon MGO stimulation on mesothelial cells. (**A**,**B**) Pathway enrichment analysis of “6 h vs. 0 h” and “24 h vs. 0 h” using the KEGG database showed that MGO initiated pathways that related with inflammation, apoptosis, cytokine–receptor response, and ECM–receptor response on MeT-5A. (**C**) DEGs involved in those significantly changed pathways were shown in the heatmap. Some DEGs were upregulated gradually along the treating time, while others were downregulated after being treated MGO for 6 h. (**D**) DEGs of cytokines and growth factors that commonly participated in inflammatory or fibrotic processes were listed.

To explore what biological processes were significantly altered upon MGO stimulation on mesothelial cells, total DEGs from comparison groups of 6 h vs. 0 h and 24 h vs. 0 h (0 h as the base) were performed for pathway enrichment analysis based on the KEGG database. Results of the 6 h vs. 0 h group showed that pathways related to TNF signaling, hypoxia-inducible factor 1 (HIF-1) signaling, and cytokine–cytokine receptor signaling were significantly changed (Figure 3A). In addition, the majority of the DEGs involved in these pathways were upregulated, indicating the activation of pro-inflammatory responses in mesothelial cells after being treated with MGO for 6 h. After mesothelial cells were treated with MGO for 24 h, the number of significantly changed pathways was increased (Figure 3B). These activated pathways were associated with pro-inflammation, cytoskeletal alteration, cytokine–receptor interaction, ECM–receptor interaction, apoptosis, and calcium signaling. After performing z-score normalization, all DEGs involved in these significantly changed pathways were illustrated in a heatmap (Figure 3C). A progressively upregulated or downregulated trend was observed on these DEGs along the treatment time. From the DEG list, genes corresponding to interleukins, chemokine ligands, and growth factors related to peritoneal fibrosis were listed (Figure 3D). Numbers of cytokine genes, such as *IL1B*, *IL6*, *IL11*, *CXCL1*, *CXCL2*, *CXCL3*, *CXCL6*, *CXCL8*, *CSF1,* and *CCL2,* that attracted leukocytes upon inflammation were upregulated after MGO treatment on mesothelial cells. Moreover, *ICAM1* and *VCAM1*, which are associated with leukocyte adhesion, were significantly upregulated after 24 h. *TLR4*, a toll-like receptor that was able to trigger the release of pro-inflammatory and fibrotic mediators [26], was also upregulated upon the stimulation of MGO (Figure 3D). Other growth factor-related genes, such as *CCN2*, *PDGFA*, *PDGFB*, *PDGFD*, and TGFβ signaling pathway-associated genes, such as *TGFB2* and *TGFBR2,* were all detected in the DEG list. Despite the fact that *TGFB1* was also upregulated, the fold change was lower than 2 when compared with non-treated cells (Figure 3D). Taken together, genes involved in pro-inflammatory and fibrosis-related pathways were observed on MGO-stimulated mesothelial cells. Since mesothelial cells were also considered to be one of the myofibroblast origins in peritoneal fibrosis [16,27], the transcriptomic switch between epithelial and mesenchymal phenotypes upon MGO stimulation was also examined. However, no solid evidence supporting mesothelial cells underwent epithelial-to-mesenchymal transition upon the stimulation of MGO can be obtained here. Marker genes of epithelial cells were not downregulated, and the changes of marker genes of mesenchymal cell markers were not unified, as some of them, such as *ACTA2*, *VIM*, and *COL1A1*, were upregulated, while other marker genes were downregulated (Supplementary Figure S2A).

### 2.4. Stimulation of MGO-Treated Mesothelial Cells Altered the Distribution of Actin Filaments on Fibroblasts

**Figure 4:**
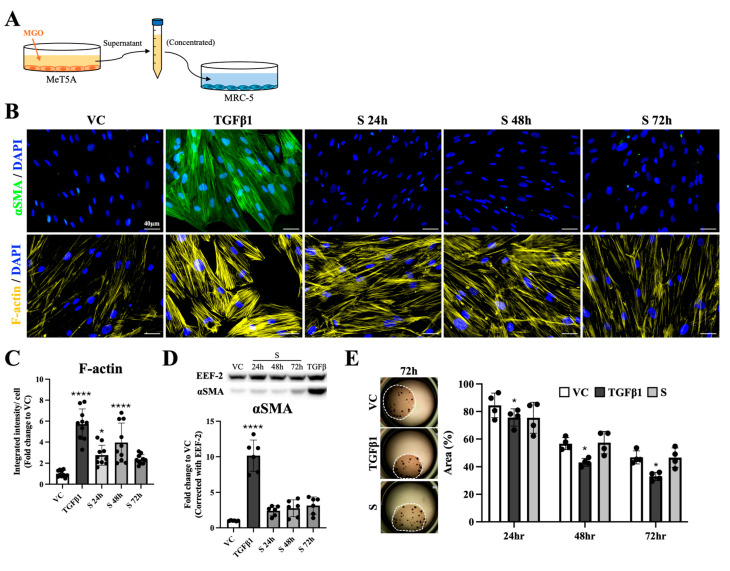
Characterization of the cytoskeleton on fibroblasts treated with supernatant generated by MGO-stimulated mesothelial cells. (**A**) Experimental setting to investigate the effects of MGO-stimulated mesothelial cells on fibroblasts. (**B**) Immunofluorescence staining showed significantly increased expression of αSMA (labeled in green) and F-actin (labeled in yellow) on cells from the TGFβ1 group. Although no signals of αSMA were observed on supernatant-treated cells, increased signals of F-actin were noted compared with cells in the VC group. (**C**) Quantification analyses showed significantly elevated signals of F-actin on cells treated with TGFβ1 and cells treated with supernatant for 24 and 48 h. (**D**) Western blot showed that fibroblasts treated with TGFβ1 expressed significantly higher αSMA, and fibroblasts treated with supernatant expressed similar αSMA with normal fibroblasts. (**E**) In the contraction assay, the area of MRC-5-laden collagen gels was significantly decreased in the TGFβ1 group, but no differences were found between the supernatant group and the VC group. Asterisks indicated significant differences between groups (**** *p* < 0.0001, * *p* < 0.05). For each experimental condition, 10 images were evaluated, and representative images were presented.

Since direct MGO stimulation did not promote fibroblast-to-myofibroblast transition, and the NGS data from MGO-treated mesothelial cells showed upregulation of multiple pro-inflammation and fibrosis mediators’ genes, whether the secretions from mesothelial cells could further activate fibroblasts was investigated. Supernatant from 2.5 mM MGO-stimulated MeT-5A was collected, concentrated, and added to MRC-5 (Figure 4A). The results from both immunofluorescent staining and Western blot demonstrated that αSMA increased significantly only in the TGFβ1-treated group but not in the VC or supernatant-treated groups (Figure 4B, upper panel, and Figure 4D). When F-actin was examined by phalloidin staining to illustrate bundles of actin filaments, which usually appeared during the activation of fibroblasts to myofibroblasts, stress fibers that described the assembly and crosslinking of actin filament bundles were prominent in the TGFβ1-treated group (Figure 4B, lower panel). As for cells treated with supernatant collected from MGO-stimulated mesothelial cells, a stick-like pattern of F-actin signals can be observed (Figure 4B, lower panel). Quantification results of fluorescence staining verified a significant increase in F-actin signals per cell after fibroblasts were treated with collected supernatant (Figure 4C). To examine whether the above-mentioned phenotypical changes corresponded to the contractility of fibroblasts, a collagen gel contraction assay was further performed. As shown in Figure 4E, only cells from the TGFβ1-treated group significantly shrank collagen gels (Figure 4E, dark gray bars), and no differences regarding the area of the gels were noted in the supernatant-treated groups (Figure 4E, light gray bars). Based on these results, fibroblasts that were treated with supernatant from MGO-stimulated mesothelial cells showed no myofibroblast phenotypes but a change in the actin filament.

### 2.5. Supernatant from MGO-Treated Mesothelial Cells Promoted Fibroblast-to-Proto-Myofibroblast Transition

**Figure 5:**
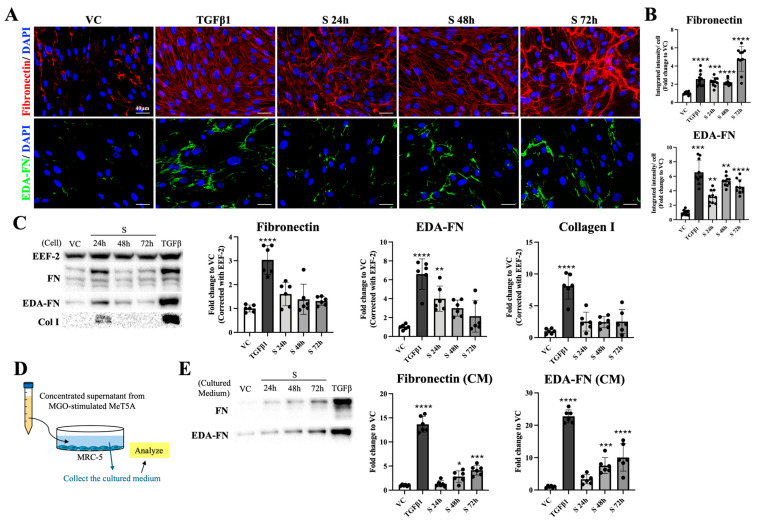
Evaluation of inter- and extracellular expression of ECM proteins on supernatant-treated fibroblasts. (**A**) Immunofluorescence staining showed increased signals of fibronectin and EDA-FN on both TGFβ1- and supernatant-treated fibroblasts. (**B**) Quantification analyses showed that both fibronectin and EDA-FN were significantly increased on fibroblasts from the TGFβ1 group and supernatant groups. (**C**) Protein expressions of fibronectin, EDA-FN, and collagen I were examined using a Western blot. For fibronectin and collagen I, elevated expressions were only observed on fibroblasts treated with TGFβ1. As for EDA-FN, fibroblasts treated with TGFβ1, as well as those treated with supernatant for 24 h, expressed significantly higher amounts than normal fibroblasts. (**D**) Illustration for samples that were used to probe fibronectin and EDA-FN in the following Western blot experiment. (**E**) The same volume of the cultured medium collected from each condition was loaded into each well of the SDS page and ran the Western blot. Significantly increased expressions of fibronectin and EDA-FN in the cultured medium were noted in the TGFβ1 group, supernatant 48 h, and supernatant 72 h group. Asterisks indicated significant differences between groups (**** *p* < 0.0001, *** *p* < 0.001, ** *p* < 0.01, * *p* < 0.05). Images from 10 different fields in each group were evaluated, and representative images were presented.

Proto-myofibroblasts, an intermediate phenotype containing EDA-FN, have been described to picture the activated fibroblasts that obtained higher ECM-secreting ability but were lacking αSMA expression. Since the supernatant-treated fibroblasts showed no expression of αSMA but significant change in actin filament, we investigated the protein expression of fibronectin, EDA-FN, and collagen I in the following experiments to see whether these fibroblasts were proto-myofibroblasts. As shown in Figure 5A, fibroblasts from TGFβ1- and supernatant-treated groups exhibited significantly increased fibronectin and EDA-FN when compared with the VC group. Quantification results also verified that fibroblasts treated with TGFβ1 or the supernatant from MGO-stimulated mesothelial cells expressed significantly higher fibronectin and EDA-FN (Figure 5B). However, although cells treated with TGFβ1 or supernatant both expressed higher signals of fibronectin, the alignment patterns of the signals were different. When fibroblasts were treated with TGFβ1, the signals of fibronectin were homogeneously increased and distributed, while disorganized stripe-like signals were observed in supernatant-treated groups (Figure 5A, upper panel; Supplementary Figure S3). As shown in Supplementary Figure S4, the expression pattern of EDA-FN, besides slight differences in the intensity, did not show obvious differences in terms of distribution pattern between the TGFβ1 group and the supernatant-treated groups. Western blot analyses from the collected fibroblasts showed that besides the expression of EDA-FN being significantly increased, protein expression of both fibronectin and collagen I was not altered (Figure 5C). To elucidate the discrepancies between the above-mentioned immunofluorescence staining and Western blotting results and to examine the localization of the increased fibronectin and EDA-FN, cultured medium from fibroblasts was collected (Figure 5D). The results showed that both fibronectin and EDA-FN were significantly increased in the TGFβ1, supernatant 24 h, and supernatant 48 h groups when compared with the VC group (Figure 5E). From the results of Figure 4 and Figure 5, fibroblasts that were treated with supernatant from MGO-stimulated mesothelial cells demonstrated stress fibers without the appearance of αSMA and an elevated ability to secrete fibronectin and EDA-FN, which is in accordance with the known characteristics of proto-myofibroblasts.

## 3. Discussion

To date, approximately 9% of kidney failure patients rely on PD to sustain their lives. Although advantages such as lower cost, higher flexibility, and similar survival rates compared with hemodialysis made PD a good option for dialysis patients, the gradual decrease in solute exchange on the peritoneum posed a significant limitation [28]. The alteration of peritoneal function with the development of ultrafiltration failure was considered to result from peritoneal fibrosis, the pathological changes in the peritoneum from long-term PD patients [29]. To cope with peritoneal fibrosis and to protect the peritoneal function during PD, elucidating the underlying mechanism is an unmet clinical need. In this study, we focused on investigating the effects of MGO on the mesothelial cells and whether MGO stimulates fibroblast-to-myofibroblast transition. Our results showed that MGO induced significant transcriptomic changes involved in pro-inflammatory, apoptosis, and fibrosis-related pathways. Inconsistent with the previous findings from in vitro studies upon the GDPs stimulation on mesothelial cells, the elevation of monocyte chemoattractant protein-1 (MCP-1), production of reactive oxygen species, and induction of apoptosis-related pathways would be observed [30,31].

The second important finding in this study was that secretions from stimulated mesothelial cells activated fibroblasts and promoted their transition into proto-myofibroblasts, an intermediate stage toward myofibroblasts. Our transcriptomic analysis (Supplementary Figure S2B) showed that not only genes responsible for promoting fibroblast-to-myofibroblast transition were upregulated, but corresponding genes for inhibiting the transition were also upregulated. The opposite effects on myofibroblast activation may partly be explained by the observed incomplete fibroblast-to-myofibroblast transition induced by mesothelial cells in our study. Besides the chemical stimuli such as cytokines and growth factors to initiate the activation of fibroblast-to-myofibroblast activation, other studies proposed that mechanical or physical stimuli such as tissue stiffness, stretching, curvature, and topography can also promote the transition [32]. Intriguingly, we observed only proto-myofibroblasts, the first stage in myofibroblast activation, in our model, indicating that mechanical stimuli or pro-fibrotic cues secreted by participating cells other than mesothelial cells might be needed to complete the transformation into myofibroblasts upon MGO-induced peritoneal fibrosis.

Apart from the stromal cells in the peritoneum, immune cells such as macrophages were also considered to play a critical role in the progression of peritoneal fibrosis [33,34]. Among infiltrated immune cells during peritoneal fibrosis, macrophages constituted over half of the portion [35]. Our NGS data from mesothelial cells showed that genes associated with recruiting macrophages (e.g., *HSPA1, HSPA2, IL6*, *HSPG2*, and *HAS3*), facilitating cell adhesion to macrophages (e.g., *ICAM1* and *VCAM1*), and producing proteins that could drive the polarization of macrophages were upregulated after MGO stimulation (Supplementary Figure S2C). In addition, the number of upregulated DEGs related to facilitating M1 polarization of macrophages was more than DEGs that were known to facilitate M2 polarization, indicating the tendency of mesothelial cells on the induction of M1 polarization after MGO stimulation. It is known that along the progression of peritoneal fibrosis, M1 and M2 macrophages both participated and may switch the characteristics at different time points [35,36,37]. In rodent studies, depletion of macrophages attenuated fibrotic injury of the peritoneum induced by dialysate. The reperfusion of M1 macrophages on these macrophage depletion mice aggravated peritoneal damage more than mice that were reperfused with M2 macrophages [38]. Furthermore, a few studies also found that direct co-culture of mesothelial cells with macrophages enhanced the protein expression of interleukin 1-beta (IL1β), TNFα, TGFβ1, and αSMA on mesothelial cells, indicating that interactions between mesothelial cells and macrophages were crucial in the development of peritoneal fibrosis [18,39].

**Figure 6:**
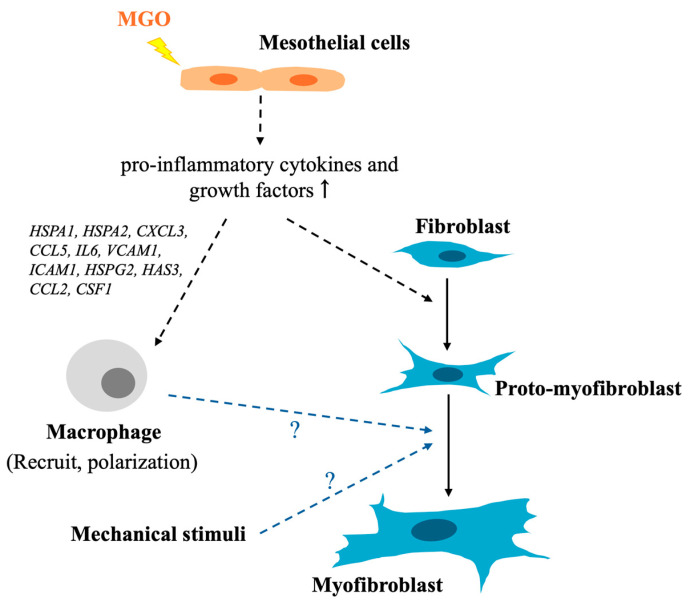
Summarized illustration of the findings in this study. Upon the administration of MGO, genes involved in pro-inflammatory and fibrotic pathways were significantly upregulated in mesothelial cells. Then, the secretions from these stimulated mesothelial cells further activated the fibroblasts into proto-myofibroblasts, which were distinct from αSMA+ myofibroblasts but also demonstrated an increase in stress fibers and elevated ECM production ability. Upregulated genes being responsible for recruiting macrophages and promoting M1 polarization were also noted and indicated a possible role for macrophages to complete the activation of myofibroblasts.

In summary, this study showed that under the stimulation of MGO, activation of signaling pathways associated with apoptosis, pro-inflammatory cytokine secretion, and fibrosis was observed in mesothelial cells. The secretions from injured mesothelial cells further activate the fibroblasts into proto-myofibroblasts, which is demonstrated by the presence of stress fibers and higher ECM production ability without αSMA when compared with fibroblasts. Genes related to recruiting macrophages and promoting M1 polarization were also upregulated, which indicated that macrophages might be one of the missing pieces to complete the transition from fibroblasts into myofibroblasts in our in vitro model (Figure 6). Apart from macrophages, additional mechanical stimuli that came from the existing sub-mesothelial stroma layer in the peritoneum may be another critical factor during the myofibroblast formation. Although the current study exhibited limitations, such as MGO rather than multiple GDPs being administrated, cell lines rather than primary cells were used. This is still, to our best knowledge, the first study to propose fibrotic tendency effects from mesothelial cells on fibroblasts under the stimulation of GDPs and a new regulatory role of mesothelial cells in the development of MGO-induced peritoneal fibrosis. The findings from this study also indicated that mesothelial cells might be a critical therapeutic target for treating peritoneal fibrosis in clinical patients. If the integrity of the mesothelial cells or the involvement of immune cells is critical for initiating and completing fibroblast to myofibroblast transition, uncovering detailed components from stimulated mesothelial cells that subsequently activate fibroblasts and targeting processes that could promote mesothelial cell regeneration would be needed for the future development of novel therapies in targeting peritoneal fibrosis.

## 4. Materials and Methods

### 4.1. Cell Culture and In Vitro Injury Model

Human mesothelial cell line (MeT-5A, # CRL-9444™), and human fibroblast cell line (MRC-5, #CCL-171™) were obtained from ATCC (Manassas, VA, USA). MeT-5A was cultured in Medium 199 (Gibco, Waltham, MA, USA) supplemented with 5% fetal bovine serum, 3.3 nM epidermal growth factor (EGF) (Sigma, St. Louis, MO, USA), 400 nM hydrocortisone (H0888, Sigma, St. Louis, MO, USA), 870 nM insulin (91077C, Sigma, St. Louis, MO, USA), and Antibiotic Antimycotic Solution (A5955, Sigma, St. Louis, MO, USA). MRC-5 was cultured in Eagle’s minimum essential medium (M4655, Sigma, St. Louis, MO, USA) supplemented with 10% fetal bovine serum, non-essential amino acids (11140-050, Gibco, Waltham, MA, USA), and antibiotic antimycotic solution (A5955, Sigma, St. Louis, MO, USA). For experiments detecting the genetic and proteomic changes, 2.5 mM and 0.5 mM MGO diluted in serum-free medium were used to treat MeT-5A and MRC-5, respectively. For experiments investigating the effects of injured MeT-5A exerted on MRC-5, MeT-5A was cultured and treated with 2.5 mM MGO for 24 h. The cultured supernatant was collected, centrifuged to remove cell debris, and filtered with a 0.22 µm filter before further concentration procedures using an ultrafiltration centrifugal tube (Macrosep ^®^, PALL, Port Washington, NY, USA) (3260× *g*, 120 min). Processed supernatant was subsequently added at a one-to-one dilution with the serum-free culture medium for MRC-5. Serum-free medium and 10 ng/mL transforming growth factor beta 1 (TGFβ1) (100-21, Peprotech, Cranbury, NJ, USA) in serum-free medium were used to treat MRC-5 as the vehicle control (VC) and positive control (PC) groups, respectively.

### 4.2. Cell Viability Assay

To determine the appropriate non-toxic concentration of MGO, the cytotoxicity effects of MGO were measured by thiazolyl blue tetrazolium bromide (MTT) assay (M5655, Sigma, St. Louis, MO, USA). Cells were seeded in 96-well plates at a density of 2 × 10^4^ cells per well and grew for 24 h until a homogenous monolayer confluency was observed. Prior to the treatments of MGO, cells were starved for 2 h. At the endpoint of the treatment, the medium was replaced with MTT solution (100 μL/well; 0.5 mg/mL) and incubated for 4 h at 37 °C. The unbound MTT was removed before DMSO (100 μL/well) (C6295, Sigma, St. Louis, MO, USA) was added and incubated for a further 1.5 h to dissolve the crystals. Optical density was measured at a wavelength of 570 nm, and background values were measured at 650 nm with a microplate reader (SpectraMax M5, Molecular Devices, Silicon Valley, CA, USA).

### 4.3. Cellular Reactive Oxygen Species Detection

To detect MGO-induced reactive oxygen species production, a 2′,7′-dichlorodihydrofluorescein diacetate (DCFH-DA) assay kit (Ab113851, Abcam, Cambridge, UK) was performed. Cells were seeded, starved for 2 h, and treated with 2.5 mM MGO for designed time points. After the treatments, cells were incubated with 25 µM DCFH-DA at 37 °C for 30 min in the dark. After washed with dPBS, some cells were directly visualized under Olympus IX83 epifluorescence microscopy (Olympus, Tokyo, Japan) to ensure the success of the staining. The other cells were trypsinized, resuspended with dPBS, and analyzed with FACScalibur^TM^ flow cytometry (BD biosciences, Franklin Lakes, NJ, USA) for quantitative analysis. Data obtained from the flow cytometry were further processed and analyzed with BD CellQuest Pro Software (Version 5.1).

### 4.4. Indirect Immunofluorescence Staining

The cells were fixed with 4% paraformaldehyde (15710, EMS, Hatfield, PA, USA) for 1 h and permeabilized with 0.5% Triton-X for 5 min at 4 °C. Non-specific signals were minimized with 1% BSA for 60 min at room temperature (RT). Primary antibody incubation was carried out with O/N incubation at 4 °C; secondary antibody incubation was carried out for 1.5 h at RT. Cell nuclei were counterstained with the mounting medium in the presence of diamidino-2-phenylindole (Ab104139, Abcam, Cambridge, UK). Negative control was performed by the same staining procedure but omitting the primary antibody. Staining results were evaluated with Olympus IX83 epifluorescence microscopy and analyzed with ImageJ software 1.53k. Background subtraction was performed identically for all images (including control images).

### 4.5. Western Blotting

An equivalent amount of protein extract (μg) was resuspended with an appropriate volume of lithium dodecyl sulfate sample buffer (NuPAGE™, Thermo Fisher Scientific, Waltham, MA, USA) in the presence of sample reducing agent (NuPAGE™, Thermo Fisher Scientific, Waltham, MA, USA). Samples were heated in a 100 °C dry bath for 10 min and air-cooled to RT before loading on gels. The Bio-Rad Mini-PROTEIN^®^ electrophoresis system was used (Bio-Rad Laboratories Ltd., Hertfordshire, UK), and standard manufactory protocol was followed. Proteins were separated by 6–10% sodium dodecyl sulfate-polyacrylamide gel (SDS-PAGE) and wet-blotted onto a polyvinylidene difluoride (PVDF) membrane (Immobilon-P, Millipore, Burlington, MA, USA). After blocking for one hour with Tris-buffered saline-Triton X100 (5 mM Tris, 250 mM sucrose, pH 7.4 with 0.05% *v/v* Tween-20 [TBST], supplemented with 5% milk powder) at RT, blots were incubated with primary antibodies at 4 °C overnight at RT for one hour. After three times washing in TBST, a secondary antibody was subsequently added, and blots were incubated at RT for another hour. After rinsing with TBST, a specific protein signal was visualized by chemiluminescence (PK-NEL122, Blossom Biotechnologies Inc.) and detected under the ChemiDoc™ XRS+ system (Bio-Rad Laboratories, Hercules, CA, USA). The relative intensity of each band was semi-quantified using ImageJ software. When necessary, blots were stripped with stripping buffer (#46430, Thermo Fisher Scientific, Waltham, MA, USA) and re-probed for other proteins of interest.

### 4.6. Collagen Gel Contraction Assay

MRC-5 was mixed with type I collagen (3.89 mg/mL; 354236, Corning, Corning, NY, USA) to reach a final concentration of 1.5 mg/mL of collagen and 10^5^ cells/mL. Collagen-MRC5 gel mixtures were loaded into 24-well plates and solidified for 1 h at 37 °C. The gel was carefully dissociated from the well by running the tips along the gel edges. Serum-free medium or 10 ng/mL TGFβ1 (diluted in serum-free medium) was given to gels of the VC or PC group, respectively. 0.5 mM MGO (diluted in serum-free medium) or supernatant collected from cultured MeT-5A (one-to-one dilution to serum-free medium) was given to collagen-MRC5 gel mixtures as the experimental groups. After treating for 24, 48, and 72 h, the MRC-5-laden collagen gels were observed under the Olympus SZ61 microscope (Olympus, Tokyo, Japan); the percentage of the gel to the whole area of the well was calculated by ImageJ to represent the contractile ability of the MRC-5 under different experimental conditions.

### 4.7. Next-Generation Sequencing 

MeT-5A treated with 2.5 mM MGO at time points of 0 (n = 4), 6 (n = 3), and 24 (n = 3) hours were collected and subjected to next-generation sequencing (RNAseq). Sample RNA was purified using TRIzol^TM^ reagent (Invitrogen, USA) and BCP reagent (Invitrogen, USA) according to the protocols. 1000 ng total RNA processed with the TURBO DNA-free™ Kit (AM1907, Invitrogen, Waltham, MA, USA) per sample was used to construct the mRNA-seq library by Universal Plus^TM^ mRNA-Seq with NuQuant^®^ (TECAN, Männedorf, Switzerland). To examine the concentration and quality of the mRNA-seq library, the dsDNA High Sensitivity Kit for Qubit^®^ 2.0 Fluorometer (Invitrogen, Waltham, MA, USA) and DNA 1000 assay for Agilent 2100 Bioanalyzer (Agilent, Santa Clara, CA, USA) were both performed. The indexed samples with good quality were pooled equimolarly and sequenced on Illumina NovaSeq 6000 (151 bases, paired-end sequencing) (Illumina, San Diego, CA, USA).

### 4.8. Bioinformatic Analysis

Upon obtaining raw data from NGS, the quality value (QV = −10log_10_(*Pe*), *Pe* = probability of error) of every base in each read was calculated. Bases that had QV below 20, ambiguous nucleotides, and adaptor sequences were trimmed, and then RNA-seq analysis was performed to align the reads with Genome Research Consortium human build 38 (GRCh38) using CLC Genomics Workbench v.10 software (QIAGEN, Venlo, The Netherlands). Fragments Per Kilobase of exon model per Million mapped fragments (FPKM) and information on genes were further obtained. Next, Pearson correlation was used for correlation analysis. Principal component analysis (PCA) was performed using the prcomp function from the R program, and the results were illustrated using cubemaker (https://tools.altiusinstitute.org/cubemaker/#; accessed on 22 February 2023). Volcano plots were plotted using ggplots (R package: v3.2.1). Differential expression genes (DEGs), genes that meet the conditions of fold changes > 2 and *p*-value ≤ 0.05, were selected and analyzed with pathway enrichment analysis from Kyoto Encyclopedia of Genes and Genomes (KEGG) Automatic Annotation Server (KAAS). Interested genes were selected, Z-score normalization was performed, and the results were represented with a heatmap drawn by TreeView software (v1.1.6r4).

### 4.9. Chemicals, Reagents, Antibodies

Rabbit monoclonal anti-eukaryotic elongation factor 2 (EEF2, Ab75748), rabbit polyclonal anti-alpha smooth muscle actin (αSMA, Ab5694), and mouse monoclonal anti-type I collagen (ColI, Ab6308) were obtained from Abcam (Cambridge, UK). Mouse monoclonal anti-fibronectin (610077) was purchased from BD (Franklin Lakes, NJ, USA). Mouse monoclonal anti-EDA-fibronectin (EDAFN, sc-59826) was obtained from Santa Cruz Biotechnology (Dallas, TX, USA). Anti-phalloidin (BT00044) was purchased from Cambridge Bioscience (Cambridge, UK). All secondary antibodies were purchased from Jackson ImmunoResearch Laboratories Inc. (West Grove, PA, USA).

### 4.10. Statistical Analyses

Statistical analyses were performed using GraphPad Prism 9 (GraphPad Software, San Diego, CA, USA). The statistical significance between groups was assessed by one-way ANOVA followed by the Mann–Whitney U test for a 2-group comparison. The statistical significances were marked with asterisks and expressed as follows: **** = *p* < 0.0001; *** = *p* < 0.001; ** = *p* < 0.01; * = *p* < 0.05; n.s. = *p* > 0.05. Data were expressed as the mean ± standard deviation (S.D.).

## 5. Conclusions

In conclusion, this study revealed that MGO-stimulated mesothelial cells could prompt fibroblasts into proto-myofibroblasts; however, additional involvement of other factors or cells (e.g., macrophages) may be needed to complete the transformation into myofibroblasts.

## Data Availability

The datasets used and/or analyzed during the current study are available from the corresponding author upon reasonable request.

## Supplementary Materials

The following supporting information can be downloaded at: https://www.mdpi.com/article/10.3390/ijms26020813/s1.

## Abbreviations

αSMA	Alpha-smooth muscle actin
DCFH-DA	2′,7′-dichlorodihydrofluorescein diacetate
DEG	Differential expression gene
ECM	Extracellular matrix
EDA-FN	Extra domain A containing fibronectin
GDP	Glucose-degradation product
IL-17	Interleukin-17
KEGG	Kyoto Encyclopedia of Genes and Genomes
MCP-1	Monocyte chemoattractant protein-1
MGO	Methylglyoxal
MTT	Thiazolyl blue tetrazolium bromide
NGS	Next-generation sequencing
PCA	Principal component analysis
PD	Peritoneal dialysis
RT	Room temperature
TGFβ1	Transforming growth factor beta 1
TNF	Tumor necrosis factor
