# Supplementary material for: Methylglyoxal-Stimulated Mesothelial Cells Prompted Fibroblast-to-Proto-Myofibroblast Transition
Source: Int J Mol Sci. 2025 Jan 19;26(2):813. doi: 10.3390/ijms26020813 (PMC11766140; doi:10.3390/ijms26020813)
Supplement: Supplementary file 1 [file ijms-26-00813-s001.zip › ijms-3420236-supplementary.pdf]

Supplementary figure legends

Supplementary figure 1

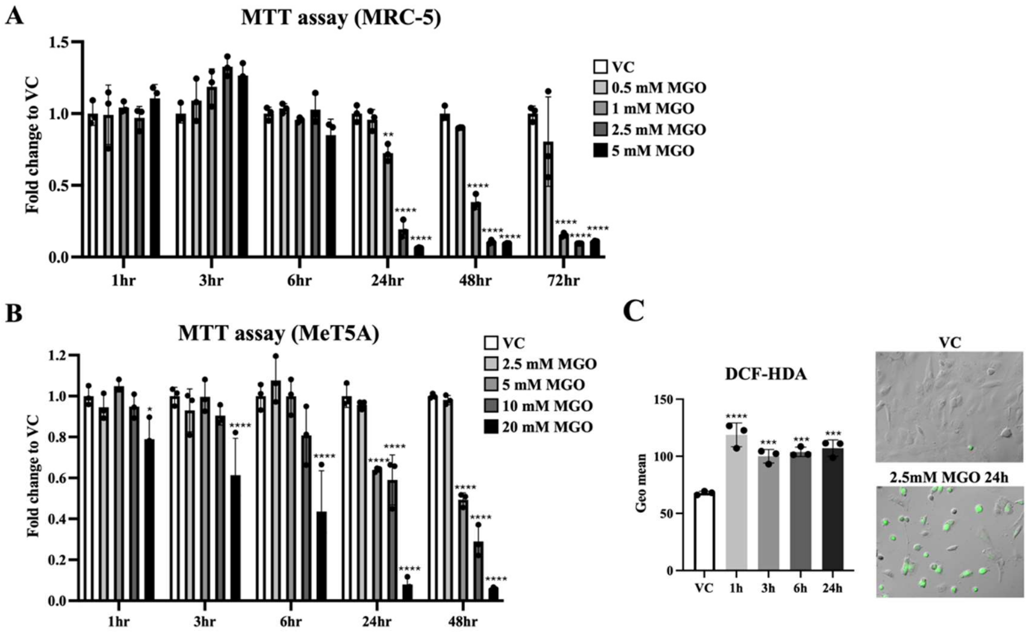

Supplementary figure S1. Cytotoxicity and reactive oxygen species production from MGO treatments. (A) It showed a significant decrease in cell viability treated with MGO beyond 1mM after 24 hours on MRC-5. (B) It was observed that the cell number of MeT-5A was significantly decreased after 1-hour treatment of 20 mM MGO. After 24 hours, number of cells that treated with MGO at concentrations beyond 5 mM were significantly dropped. (C) DCF-HDA assay showed a significant elevation in reactive oxygen species production after 1-hour treatment of 2.5 mM MGO on mesothelial cells. Asterisks indicated significant differences between groups (****p < 0.0001, ***p < 0.001, **p < 0.01, *p < 0.05, ns not statistically different). For each experimental

condition, 3 biological replications were performed. For flow cytometry results of DCF-HDA assay, 10^5 cells were evaluated in each sample.

Supplementary figure 2

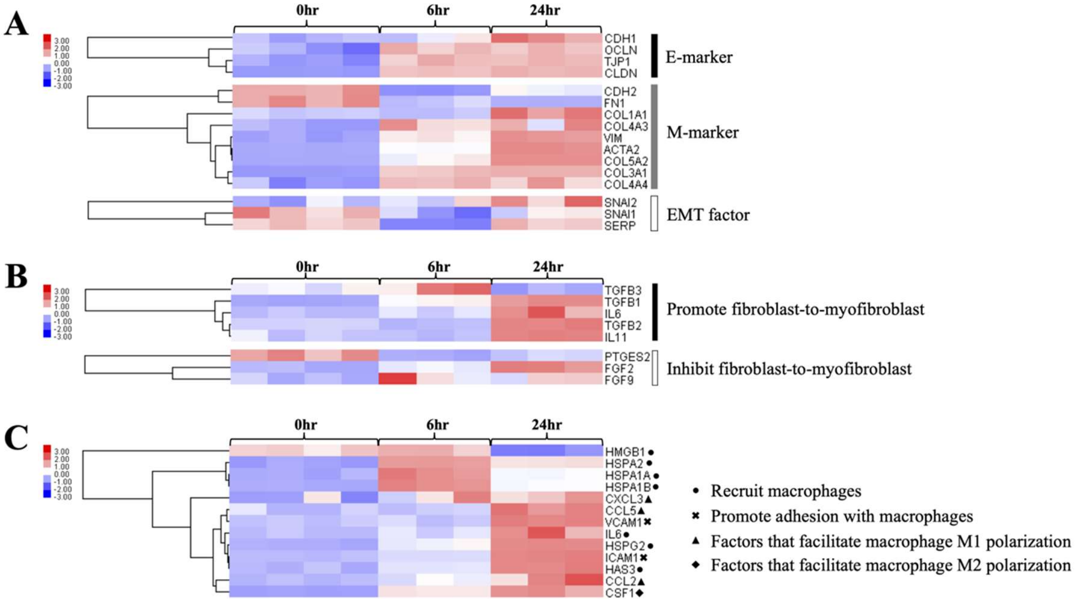

Supplementary figure S2. Gene sets associated with epithelial-to-mesenchymal transition, myofibroblasts activation, and interactions with macrophages on MGO-treated MeT5A. (A) Heatmap regarding genes of epithelial cell marker (black line), mesenchymal cell marker (grey line), and epithelial-to-mesenchymal transition (EMT) inducing factors (white line) showed no trend of EMT on MGO-treated mesothelial cells. (B) Genes being responsible for proteins promoting (black line) or inhibiting (white line) the fibroblast-to-myofibroblast transition and their relative expression among different time points were plotted in a heatmap. (C) DEGs related to recruiting macrophages, promoting adhesion with macrophages, or secreting factors that could facilitate M1 or M2 polarization on macrophages were plotted.

Supplementary figure 3

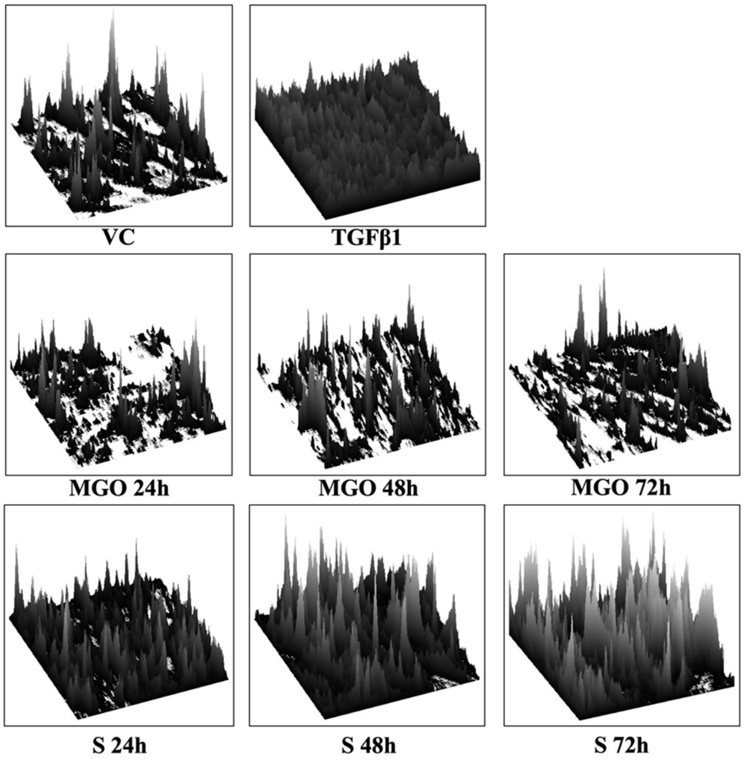

Supplementary figure S3. Surface plots of immunofluorescent signals of fibronectin.

The expression patterns of fibronectin in immunofluorescence staining under different experimental conditions were depicted in surface plots. It was noted that distributed patterns of cells in VC group and MGO-treated groups were quite similar. Though increased fluorescent signals of fibronectin were both observed in TGFβ1 and supernatant-treated groups while their distribution was different. The pattern from TGFβ1 group was more homogeneously distributed while that from supernatant-treated groups were more disorganized.

Supplementary figure 4

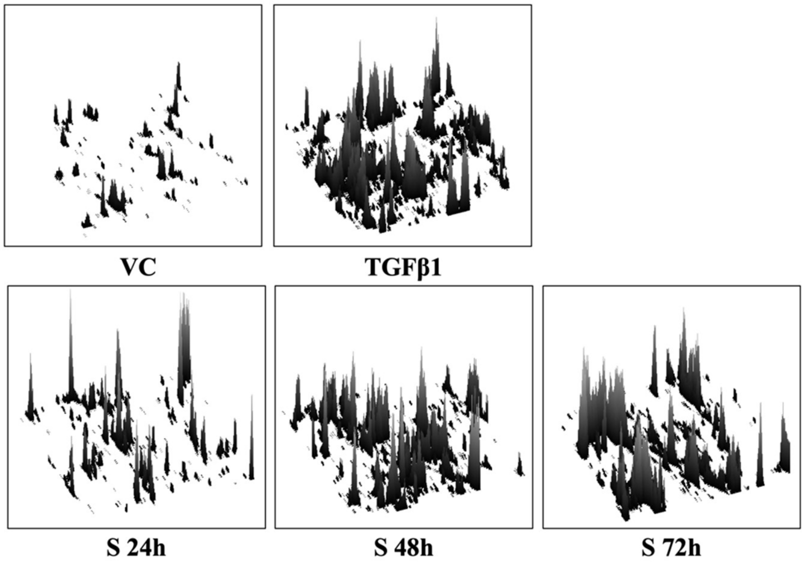

Supplementary figure S4. Surface plots of immunofluorescent signals of EDA-FN.

The expressed patterns of EDA-FN in immunofluorescence staining under treatment of TGFβ1 and supernatant from MGO-stimulated mesothelial cells were depicted in surface plots. It was observed that TGFβ1- and supernatant-treated groups showed increased expression of EDA-FN and the expressed patterns were quite similar.
